# Infantile Œdema of the Lower Limbs

**Published:** 1907-12-28

**Authors:** 


### Diseases of Children.

### INFANTILE ŒDEMA OF THE LOWER LIMBS.

The common cause of œdema of the feet and legs in infancy is the malnutrition resulting from chronic wasting disorders, such as tuberculosis, syphilis, prolonged alimentary affections, and any cause of marasmus. Sometimes it is due to toxæmia or to renal mischief. Occasionally it follows overfeeding after a period of starvation, *e.g.* the œdema seen after operations for the relief of congenital stenosis of the pylorus.

### General Points.

But there is another class of cases for which none of the above explanations holds good, cases which appear to be truly congenital in development and hereditary in origin. Milroy, of Omaha,* described a solid œdema which affected 22 out of 97 members of a family in six generations. Twelve were males and seven females. It affected one or both legs and was congenital, except in one child, in whom it began at the age of 12 years. Rolleston had recorded similar cases, each of three years' duration, in a boy aged 13 years and a girl aged 16 years. Their mother also had been affected since the age of ten years, but five other children had escaped. Isolated cases similar in nature but with no hereditary history have been recognised. The œdema has been reported as a sequel of measles, in a babe of three months, affecting the left leg and face, by Crozer Griffith and Newcome. The characteristic features are as follows: One or both lower limbs, chiefly the feet and legs, are affected. The œdema increases gradually when the patients are up and about, and decreases when they are lying down. The skin is anæmic, natural in colour if the limbs are kept warm, but usually cold and pale. Pitting is induced fairly readily on pressure, and leaves a pallid area for some time after on account of the feebleness of the circulation. There is no structural change in the skin, nothing analogous to elephantiasis. Usually there is evidence of a feeble circulation, such as a history of lividity at birth, blueness of the hands and feet in cold weather, and a liability to chilblains. The blueness is increased by the action of gravity, but is not associated with pain or tenderness. It may be so marked as to suggest the lividity of local asphyxia or Raynaud's disease. Mental condition, sensation, reflexes, and bodily development are normal. There is no evidence of heart disease, no deficiency of red cells in the blood, and no history of hæmophilia, hæmoglobinuria, or local gangrene.

### A Note on its Pathology.

The pathology is still in doubt. No obstructive heart disease, venous thrombosis, or pressure on the vena cava inferior, and no lymphatic obstruction have been found, nor is there any proof of absence of valves in the veins. It may be due to lymph stasis, but the absence of skin changes is opposed to such a view. Most probably it is the result of excessive transudation, due to some inherent defect in the blood-vessels or the blood. If so, it presents analogies with hæmophilia. From another point of view we must consider its connection with angio-neurotic œdema, another hereditary disease liable to affect many members of a family. At all ages, perhaps most frequently in later childhood and about puberty, the hands and feet, especially the fingers and toes, may present an appearance of swelling, coldness, and more or less lividity, with great liability to chilblains. The special, but unnecessary, name of "acro-cyanosis" has been devised for this condition, which is in all probability dependent on vaso-dilatation. Both in boys and girls it may be associated with orthostatic or functional albuminuria, and both these symptoms may be caused by that vague state of the nervous system known as neurasthenia. Such a condition cannot be mistaken for the infantile œdema above described, though it may suggest an analogous vascular pathology.

### Differential Diagnosis.

But there is a variety of œdema of the feet in infants which may be mistaken for the hereditary affection or ascribed to a similar pathology, if there is no family history of the complaint. Occasionally one sees infants with a fairly solid œdema of the feet, affecting both dorsal and plantar surfaces, involving the toes, and perhaps extending above the ankles. It is associated with coldness and lividity, if the weather is cold. The mother may state that it has been present since birth, or that it varies from time to time, sometimes in accordance with the amount of urine passed. Probably many of these cases depend on renal inadequacy or actual renal disease. Examination of the centrifugalised urine may yield evidence thereof, or the diagnosis may be supported by morning œdema of the eyelids or conjunctiva. Sometimes the urinalysis only yields dubious evidence in the form of a few hyaline casts and renal cells. Cases of this sort are occasionally seen in hospital out-patient practice and at medical societies, but the ultimate result and the pathological anatomy require investigation.

Hereditary œdema is chiefly of importance from a prognostic, and consequently a diagnostic, point of view. It is persistent and harmless, but troublesome on account of the weight and clumsiness of the limbs, and because of the coldness and liability to chilblains. Possibly it delays development by mechanical interference with exercise.

### Treatment.

Little can be done in the way of treatment. Bandages give some support, but the œdema appears above the bandages. Rest and elevation of the limb afford partial or temporary relief from the swelling. The general health should be maintained, and an attempt may be made to improve the vascular tone by the use of ergot, digitalis, and galvanism. Massage in the upward direction, the course of circulation, is of value, and the limbs should be kept warm.

* "New York Medical Journal," November 5, 1904.

